# Structural Implications of Genotypic Variations in HIV-1 Integrase From Diverse Subtypes

**DOI:** 10.3389/fmicb.2018.01754

**Published:** 2018-08-02

**Authors:** Leonard Rogers, Adetayo E. Obasa, Graeme B. Jacobs, Stefan G. Sarafianos, Anders Sönnerborg, Ujjwal Neogi, Kamalendra Singh

**Affiliations:** ^1^Christopher S. Bond Life Sciences Center, University of Missouri, Columbia, MO, United States; ^2^Division of Medical Virology, Department of Pathology, Faculty of Medicine and Health Sciences, Stellenbosch University, Stellenbosch, South Africa; ^3^Division of Clinical Microbiology, Department of Laboratory Medicine, Karolinska Institute, Stockholm, Sweden; ^4^Laboratory of Biochemical Pharmacology, Department of Pediatrics, Emory University School of Medicine, Atlanta, GA, United States; ^5^Division of Infectious Diseases, Department of Medicine Huddinge, Karolinska Institute, Stockholm, Sweden; ^6^Department of Molecular Microbiology and Immunology, University of Missouri, Columbia, MO, United States

**Keywords:** HIV-1 integrase, strand transfer inhibitor, polymorphism, drug-resistance, HIV-1 subtypes

## Abstract

Human immunodeficiency virus type 1 (HIV-1) integrase (IN) integrates viral DNA into the host genome using its 3′-end processing and strand-transfer activities. Due to the importance of HIV-1 IN, it is targeted by the newest class of approved drugs known as integrase strand transfer inhibitors (INSTIs). INSTIs are efficient in maintaining low viral load; however, as with other approved antivirals, resistance mutations emerge in patients receiving INSTI-containing therapy. As INSTIs are becoming increasingly accessible worldwide, it is important to understand the mechanism(s) of INSTI susceptibility. There is strong evidence suggesting differences in the patterns and mechanisms of drug resistance between HIV-1 subtype B, which dominates in United States, Western Europe and Australia, and non-B infections that are most prevalent in countries of Africa and Asia. IN polymorphisms and other genetic differences among diverse subtypes are likely responsible for these different patterns, but lack of a full-length high-resolution structure of HIV-1 IN has been a roadblock in understanding the molecular mechanisms of INSTI resistance and the impact of polymorphisms on therapy outcome. A recently reported full-length medium-resolution cryoEM structure of HIV-1 IN provides insights into understanding the mechanism of integrase function and the impact of genetic variation on the effectiveness of INSTIs. Here we use molecular modeling to explore the structural impact of IN polymorphisms on the IN reaction mechanism and INSTI susceptibility.

## Introduction

Combination antiretroviral therapy (cART) targets several steps of viral replication. In many cases, cART can suppress viral load below the detection level and make HIV infection a chronic yet manageable disease with a near-normal life expectancy (Antiretroviral Therapy Cohort Collaboration, 2017). However, use of cART has been constantly challenged by the emergence of both acquired and transmitted drug resistance mutations (DRMs). In addition, anti-HIV drugs have associated toxicity and bioavailability issues, although to different extents. These challenges have spurred the development of new antiretrovirals that have a high genetic barrier to resistance and low toxicity and that are effective against resistant viruses. Integrase strand transfer inhibitors (INSTIs) are the newest class of approved anti-HIV drugs. As the name implies, INSTIs inhibit HIV-1 integrase (IN), which is one of the three enzymes encoded by the *pol* gene.

Retrovirus INs have two major catalytic activities: a 3′-end processing (3′EP) that excises a dinucleotide at the 3′-end, and a strand transfer (ST) activity that integrates HIV DNA into the host chromosome. Both activities are conducted by the same active site (Craigie, 2001, 2012; Chiu and Davies, 2004). In addition, two more IN associated activities have been reported. These are (i) specific endonucleolytic cleavage at the terminal sequences of each LTR, and (ii) disintegration, which can be considered as the reverse of the ST reaction (Thierry et al., 2016).

HIV-1 IN, a 32 kDa protein, functions as a tetramer and/or higher-order oligomer (Craigie, 2012; Craigie and Bushman, 2012). HIV-1 IN has three distinct domains: the N-terminal domain (NTD) (residues 1–46), the catalytic core domain (CCD) (residues 56–186), and the C-terminal domain (CTD) (195–288). The active site (D64, D116, and E152) resides in the CCD. The NTD contains a conserved Zn^2+^-binding motif (His-His-Cys-Cys). The CTD is basic in nature and adopts an SH3-fold. The CTD has been implicated in DNA binding and oligomerization of HIV-1 IN (Kessl et al., 2009). The high resolution structure of full-length HIV-1 IN is not known (Craigie, 2012), although structures of IN domains (NTD/CCD and CCD/CTD) have been determined (Cai et al., 1997; Chen et al., 2000; Wang et al., 2001).

Currently used first-line cART includes an INSTI in the backbone of two nucleoside reverse transcriptase inhibitors (NRTIs). At present, four INSTIs have been approved. These are raltegravir (RAL), elvitegravir (EVG), dolutegravir (DTG), and bictegravir (BIC). Of these, RAL and EVG are referred to as first-generation, whereas DTG and BIC are second-generation INSTIs (Anstett et al., 2017). Another INSTI, cabotegravir (CAB), is in the late phases of clinical trials, and it has shown great potential, especially as a long-acting (LA) antiviral (Whitfield et al., 2016). Resistance pathways to first-generation INSTIs and DTG are well established. However, the same for CAB and BIC are not well understood. Molecular details of resistance to INSTIs are also not well known, mostly due to the lack of a high-resolution full-length structure of HIV-1 IN.

Recently, great strides have been made in the structural biology of INs. CryoEM structures of the HIV-1 intasome were reported in early 2017 (Passos et al., 2017). These structures, together with a 3.8 Å resolution X-ray crystal structure of the Rous sarcoma virus (RSV) intasome (Yin et al., 2016), the cryoEM structures of the prototype foamy virus (PFV) intasome (Ballandras-Colas et al., 2017), and that of the mouse mammary tumour virus (MMTV) intasome (Ballandras-Colas et al., 2016), provide significant insights into the assembly of retroviral IN nucleoprotein complexes at different stages of the ST reaction (Engelman and Cherepanov, 2017). These structures reveal many common features of the IN mechanism of action across retroviruses (Engelman and Cherepanov, 2017). In addition, the crystal structures of PFV INs in complex with DNA and DNA/INSTI have been solved (Maertens et al., 2010; Hare et al., 2010a, 2012). The crystal structures of PFV IN in complex with DNA and INSTIs have been serving as the model systems for understanding INSTI resistance mechanisms.

Limited solubility of HIV-1 IN in buffers containing isotonic salt concentrations has hindered structure determination of full-length HIV-1 IN. To overcome this limitation, Li et al. (2014) generated a chimera containing full-length HIV-1 IN fused with *Sulfolobus solfataricus* protein Sso7d that greatly enhanced the solubility and activity of the HIV-1 IN. This construct facilitated the solution of a cryoEM structure of the HIV-1 IN strand transfer complex (STC) intasome (Passos et al., 2017). Despite some limitations (discussed later), this structure provided the first glimpse of nucleoprotein organization that could be used to deduce the effect of a polymorphism (PM, defined as the change in genomic sequence variation that is common in more than 1% of the sequences) in the IN of different HIV-1 subtypes at the atomic level. Here we present the identification of IN PMs from diverse HIV-1 subtypes and assess the impact of these PMs in the structure-function mapping of HIV-1 IN.

## Materials and Methods

### Identification of PMs From Diverse HIV-1 Subtypes

HIV-1 integrase sequences (*n* = 8114) of viruses isolated from individual patients were downloaded from the HIV-1 Stanford Database (Rhee et al., 2003). After excluding the low quality and shorter sequences, we included HIV-1 A1/A2 (*n* = 483), HIV-1B (*n* = 4379), HIV-1C (*n* = 1155), CRF01_AE (*n* = 1581), and CRF02_AG (*n* = 522) sequences. These five subtypes and Circulating Recombinant Forms represent >90% of global infections (Hemelaar, 2012). Multiple sequence alignment was performed using ClustalX (Larkin et al., 2007) against the HIV-1 HXB2 sequence (the reference sequence). Variant calling of each residue was performed using an in-house R script (Krzywinski et al., 2009). We also used the IN sequences (*n* = 91) from the Tygerberg Virology (TV) cohort (Jacobs et al., 2009). This cohort contains treatment-naïve patients from a variety of ethnic groups and sexual orientations. The patient samples of the TV cohort were collected between 2000 and 2001, before the initiation of South Africa’s national HIV treatment program. Recently, we amplified and sequenced IN genes from the TV cohort (*n* = 91) for identification of INSTI DRMs (Brado et al., 2018). Subtype specific consensus sequences were generated using the Consensus Maker^1^ tool. Naturally occurring polymorphisms (PMs) were defined as any mutations that were present in >50% of sequences.

### Structures of IN Intasomes From Different Subtypes

Homology-derived molecular models of HIV-1 IN tetramers from different subtypes were generated using the cryoEM structure of the HIV-1B IN intasome (PDB file 5U1C) (Passos et al., 2017) as a template, using Prime (version 4.2) through Maestro (Schrodinger, New York, NY, United States) as previously described (Haggblom et al., 2016). The homology models were minimized using Prime and MacroModel to eliminate steric overlaps and to optimize sidechain conformations, respectively (Singh et al., 2012). The cryoEM intasome structure (PDB file 5U1C) is missing residues A205 to N222 (Passos et al., 2017). In addition, residues 1–55 (comprising the entire NTD and the linker region between the NTD and CCD), residues 135–150, and residues 186–195 could not be resolved in the outer IN molecules (definition of outer and inner INs presented below). These loops were constructed with Prime’s loop modeling utility (Schrödinger Suite). All modeled structures were submitted to the Structure Analysis and Verification Server (SAVES)^2^ as well as the Protein Structure Preparation tool of SYBYL-X (version 2.1). No bad contacts were noted in the final models of INs. The backbone torsion angles were checked by Ramachandran plot for allowed conformations of φ and ψ angles.

## Results and Discussion

### IN PMs

**FIGURE 1:**
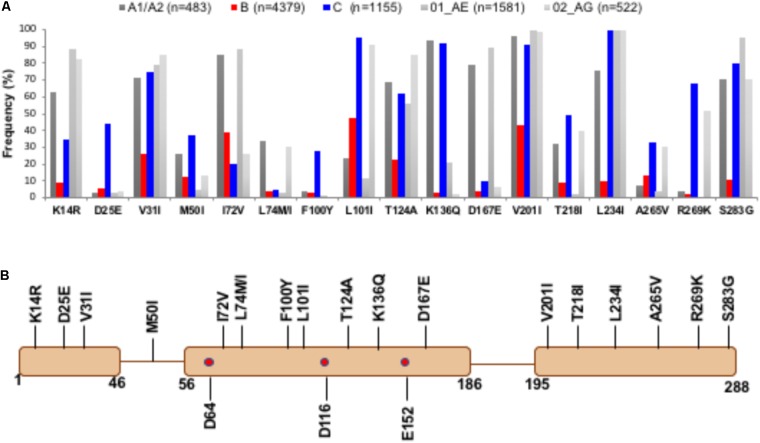
Prevalence of PMs in IN genes from diverse subtypes – Panel **A** shows the quantitative distribution (frequency in percentage) of naturally occurring polymorphisms in HIV-1C (in blue) and A-like (A1/A2, 01_AE, and 02_AG, in shades of gray) which are statistically significantly different compared to HIV-1B (shown in red). Panel **B** shows the positions of PMs in the context of IN domains. The active site residues (D64, D116, and E152) are marked as red dots.

Among all non-B subtype sequences, 17 naturally occurring IN PM positions with 18 PMs were observed (**Figure 1A**). Distribution of these PMs in different domain contexts is depicted in **Figure 1B**. Fifteen of these PMs were successfully mapped structurally. Three of these (K14R, D25E, and V31I) belong to the NTD, whereas M50I belongs to the loop region connecting the NTD and CTD. Eight PMs (I72V, L74M/I, F100Y, L101I, T124A, K136Q, and D167E) are part of the CCD, and the remaining six (V201I, T218I, L234I, A265V, R269K, and S283G) belong to the CTD. The other two were in regions for which structural data are unavailable. IN PM T218I lies within the missing region (residues 205–222). While we have modeled this region as a loop, an ambiguity remains in the secondary structure assignment, as this region in the crystal structure of CCD + CTD assumes a helical conformation. A polymorphism at position 283 could not be structurally mapped, since the structure of the C-terminal end of IN (amino acids 270–288) is unavailable.

### Structure of HIV-1 Intasome

**FIGURE 2:**
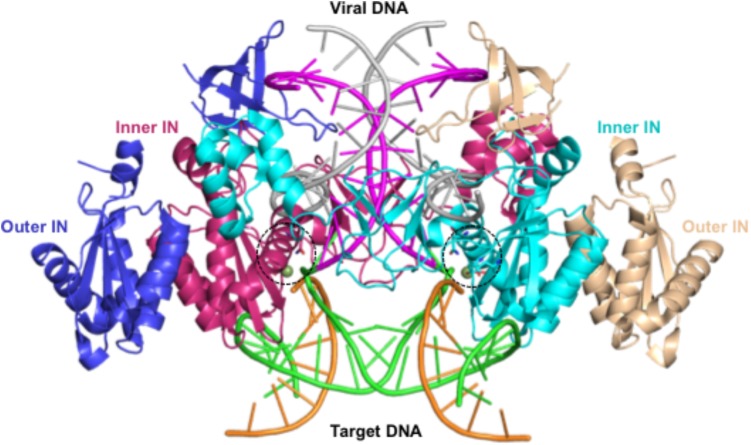
Structure of HIV-1B intasome – This figure shows the structure of the HIV-1 synaptic stable complex (SSC) intasome as determined using cryoEM (PDB file 5U1C). This structure represents the complex after the strand transfer reaction. The integrated viral DNA is rendered as ladders in magenta and gray colors, whereas the host DNA is colored light green and orange. Two IN molecules that directly bind to DNA are shown as ribbons in cyan and maroon. These IN molecules are referred to as inner INs. The other two IN molecules that bind to inner INs are rendered as ribbons in medium blue and wheat colors. These IN subunits are called outer IN molecules.

The tetrameric cryoEM structure represents a post-catalysis synaptic STC of the HIV-1 intasome formed by four IN subunits arranged in two-fold symmetry (Passos et al., 2017). A pair of IN dimers encapsulates the viral/host DNA chimera, in which the two inner molecules directly interact with DNA, while the outer molecules have protein–protein interactions with the inner molecules (**Figure 2**). This arrangement of IN oligomers bound to the viral/host DNA chimera has been referred to as the intasome core structure (Engelman and Cherepanov, 2017). The structure of the NTD in the outer IN molecules is not available in PDB coordinate file 5U1C (Passos et al., 2017). The structure of residues 205–222 is missing in all IN structures, whereas the structures corresponding to residues 135–150 and 186–195 of the outer IN molecules are also unresolved. Residues 135–139 assume a β-strand conformation in the structure of NTD+CCD [PDB file 1K6Y, (Wang et al., 2001)], whereas residues 186–195 exist as a loop structure in the crystal structure of CCD+CTD [PDB file 1EX4, (Chen et al., 2000)]. In an earlier structure of IN CCD and CTD domains, residues 205–222 are part of the helix (PDB file 1EX4). However, due to the topological position of the CCD and CTD in the STC, a loop conformation of this region appears more feasible, which is the conformation in our modeled structures. We set the conformation of residues 135–139 as a β-strand, whereas residues 186–195 were kept in loop conformation in all IN molecules.

### PMs in the NTD

**FIGURE 3:**
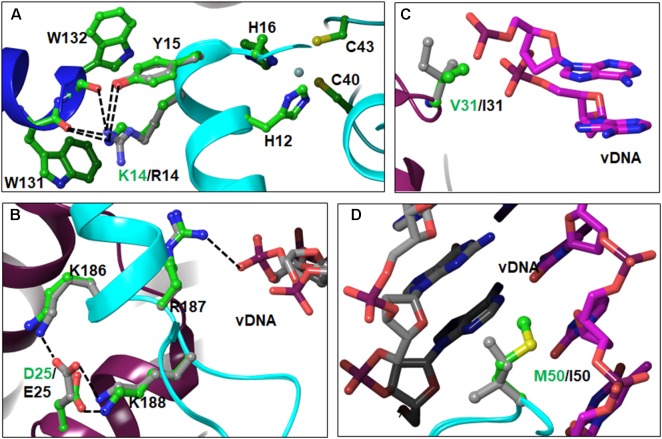
Locations and interactions of NTD PMs – Panels **A–C** show PMs and their interactions in the NTD. The M50I PM belongs to the linker region and is shown in panel **D**. The interactions (hydrogen bonding and/or ion-pairs) are shown as dotted lines. In this and following figures, the amino acid residues are shown in ball-and-stick representation and colored as green carbons for HIV-1B and gray carbons in non-B HIV-1. The other atoms are colored as red (oxygen), blue (nitrogen) and yellow (sulfur). The DNA is shown as sticks. The viral DNA is colored with magenta and gray carbons, and target DNA is colored with green and orange carbons. For reference purposes, we have included the zinc-binding motif in panel **A**.

Our analyses showed a total of three NTD PM positions in different subtypes. These are K14R, D25E, and V31I. PM M50I is located in the linker region connecting the NTD and CCD. Of these, D25E, V31I, and M50I were also noted in the cohort of South African HIV-1C patients (Brado et al., 2018). PM K14R was most prominent in CRFs 01_AE and 02_AG, followed by subtypes A1/A2. Approximately 33% of HIV-1C sequences had 14R. Amino acid K14 is located on αA-helix. The sidechain of K14 in HIV-1B forms a salt-bridge with the backbone C = O of W131 of the outer IN subunits, suggesting that K14 is involved in tetramer formation (**Figure 3A**). Substitution of K to R results in an additional salt-bridge formation (with C = O of W132). Typical salt-bridge energy ranges between 1 and 4 kCal/mol (Honig and Hubbell, 1984; Wimley et al., 1996), suggesting that R14 may induce a change in interaction energy due to a pair of salt-bridges and reduced compared to one in case of K14. In addition, both K14 and R14 form H-bonds with Y15. Previously, Y15 has been shown to be crucial for the assembly of IN and HIV-1 RT on viral RNA through the RT-IN precursor form (Takahata et al., 2017). In addition to the above mentioned interactions of K/R14, the K14 side chain forms a cation-π interaction with the indole ring of the W131 side chain, which may further stabilize the tetramer. Residue K14R is between zinc-binding residues H12 and H16. Due to the location of these residues on the α-helix, the sidechain of K14/R14 is extended away from the zinc-binding residues, and does not appear to affect the geometry of the zinc-binding motif.

PM D25E was noted exclusively in HIV-1C in the sequences from the HIV database as well as in our TV cohort (Brado et al., 2018). D25 is located at the beginning of αB-helix. D25 forms symmetrical interactions between the inner INs. D25 from one inner IN forms a salt-bridge with K188 of the other inner IN molecule (**Figure 3B**). K188 is one of the three (K186, R187, and K188) positively charged residues in the vicinity of D25. Of these, R187 directly interacts with the phosphate backbone of viral DNA (**Figure 3B**). In the modeled structure of HIV-1C IN, E25 interacts with both K186 and K188 (**Figure 3B**). Additional interactions of E25 may influence DNA binding by IN, which in turn can affect INSTI binding, since the INSTI binding pocket is formed by both DNA and protein molecules. The third PM of the NTD, V31I, exists in ∼75–85% of sequences among non-B subtypes (**Figure 1**). In the STC, V31 is close to viral DNA (**Figure 3C**), although not within interacting distance. The longer sidechain of I31 brings DNA and protein within interacting distance. Hence, the V31I PM can influence binding of DNA with IN in non-B subtypes.

Amino acid M50 is located on the linker region between the NTD and CCD. In the STC, M50 from both inner IN molecules interact with viral DNA through hydrophobic interactions with base moieties (**Figure 3D**). Substitution I50 will enhance the hydrophobic interaction due to greater hydrophobicity of I compared to M. In breakthrough selections, M50I mutations have been seen to emerge after R263K (Tsiang et al., 2016) and to provide a replication advantage to R263K-containing viruses. While M50I alone did not change the EC_50_ in seven of 24 recombinant viruses in our previous report (Neogi et al., 2018), it is possible that the M50I PM in non-B subtypes may be advantageous for R263K-containing viruses.

### PMs in the CCD

**FIGURE 4:**
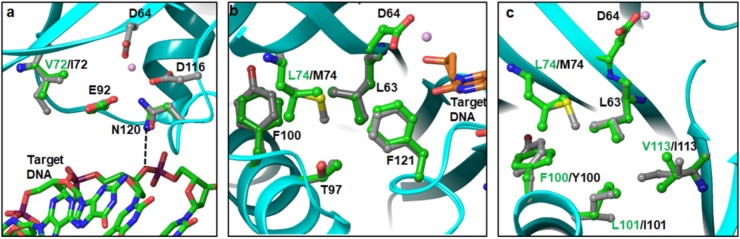
Topological positions of IN PMs near the active site of CCD – Panels **a**, **b**, and **c** show the positions of V72I, L74M and F100Y/L101I, respectively. As a reference point, D64 and metal ion at the active site are also shown.

A total of eight PMs (I72V, L74M/I, F100Y, L101I, T124A, K136Q, and D167E) in the CCD were identified in our sequence analyses. In the cryoEM intasome structure, position 72 is occupied by valine. It is not surprising, since I72 is highly polymorphic in HIV-1B (**Figure 1**). To evaluate the contribution of isoleucine, we modeled I72 in place of V72 and conducted energy minimization of the structure. Our results showed some rearrangement of neighboring sidechains including E92. Mutations E92Q/A are known DRMs against first-generation INSTIs RAL and EVG (Wensing et al., 2017). E92 is positioned between I/V72 and N120, and does not interact directly with DNA (**Figure 4a**). However, N120 has a direct interaction with the bridging phosphate oxygen of target DNA (shown as dotted line in **Figure 4a**). In the model of I72 containing IN, we noticed that the positions of the sidechain carbonyl and NH_2_ groups of N120 are flipped relative to the conformation of N120 in the V72 structure. This flipped position of the C = O requires a water-mediated contact, indicating that the I72 PM may influence emergence of the DRM at E92. To assess the effect of the E92Q/A mutation, we superposed the crystal structure of PFV IN bound to RAL and DNA (PDB file 3OYA) (Hare et al., 2010b) onto the structure of the HIV-1 intasome (PDB file 5U1C) (Passos et al., 2017). This superposition showed that at least two nucleotides at the 3′ end of the 8-nt long target DNA must be displaced to accommodate the oxadiazole moiety of RAL. A second observation that we made was that there are several water molecules in the vicinity of RAL, and three of these water molecules appear to complete the coordination geometry of Mg^2+^ ions. In the superposed structure, these water molecules are within interacting distance of E92. The mutation E92Q/A is expected to disrupt the spatial arrangement of these waters which, in turn, may affect the coordination of Mg^2+^ ions and thereby result in reduced binding of RAL. L74 is part of a hydrophobic cluster including L63, T97, F100/Y100, L101, L113/I113, and F121 near the active site of IN (**Figures 4b,c**). F100Y and L101I are also highly polymorphic in our sequence and in the HIV-1 sequence database. Both T97 and F121 are known INSTI DRM positions (Wensing et al., 2017). In addition, a recent report showed that the L74F mutation increased resistance to second-generation INSTIs (Hachiya et al., 2017). Hence, the impact of PMs in this hydrophobic cluster appears rather complex. In our modeled HIV-1C IN structure, M74 is closer to T97 and F121 than L74 (**Figure 4b**). Previous *in vitro* selection studies have shown that Q148H/R and G140S in combination with mutations L74I/M, E92Q, T97A, E138A/K, G140A, or N155H are associated with 5- to 20-fold reduced DTG susceptibility (Kobayashi et al., 2011). It is possible that polymorphism L74M is related to T97A mutation evolutionarily. F100Y and L101I PMs can impact the core structure and thereby affect the local geometry of the active site. The sequence containing I101 also had I113 in our model of HIV-1C IN. It is possible that two mutations arise simultaneously for preferred replication of the virus.

**FIGURE 5:**
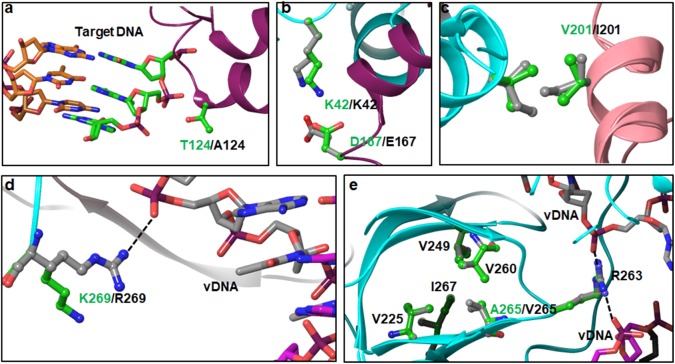
Interactions of PMs in CCD and CTD domains – Panels **a** and **b** show the position and interactions of T124A and D167E PMs of the CCD. Panels **c** and **d** show the interactions of V201I and R269K PMs of the CTD. Panels **d** and **e** show the positions of K269R and A265V PMs, respectively.

T124 interacts with target DNA (**Figure 5a**). This is a highly polymorphic position, noted here as well as reported previously (Kobayashi et al., 2011). In a previous report, the T124A mutation alone or in combination with L101I (T124A/L101I) was identified more frequently in RAL failing patients than in INSTI-naïve HIV-1B patients (Malet et al., 2011). In another study, the T124A mutation was highly prevalent in INSTI-naïve and RAL-failed patients, and it was significantly associated with HIV-nonB (Saladini et al., 2012). While the T124A mutation was selected under DTG pressure, it does not significantly affect the efficacy of DTG (Kobayashi et al., 2011; Vavro et al., 2013). These results suggest that the T124A mutation affects the binding of RAL more than the binding of DTG. T124 from the inner subunit is within interacting distance with the phosphate group bridging two nucleotides that base pair with the forth and fifth nucleotides from the 3′-end of the 8-nt long target DNA (**Figure 5a**). This interaction will be lost with T124A. It is possible that this mutation does not affect DTG binding significantly compared to RAL, as RAL is a larger molecule due to the presence of an oxadiazole ring. In the outer subunits, T124 is exposed at the surface. Hence, the implication of T124A on other viral functions such as viral fitness cannot be deduced from available structures. From the modeled structures, the interactions of K136Q are difficult to determine for two reasons: (i) the position of K136 in the outer molecules of 5U1C is not known, and (ii) K136 is near the segment that is missing in the cryoEM structure (205–222). Nonetheless, one can speculate that K136 in the inner molecule interacts with residues in the region 205–222. It should also be pointed out here that residues in the 205–222 region in the crystal structure of CCD+CTD (PDB file 1EX4) (Chen et al., 2000) assume a helical conformation, whereas in the HIV-1 intasome structure (PDB file 5U1C) (Passos et al., 2017), they form an unordered structure that appears close to K126/Q136. Amino acid D167 from one inner IN molecule interacts with K42 of the other inner IN molecule (**Figure 5b**). This is a symmetric interaction similar to that seen for the D25E polymorphism. Due to the longer sidechain of E167 compared to that of D167, there are two interactions with E167, which contributes to additional stability of the intasome tetramer in non-B.

### PMs in the CTD

There are six PMs (V201I, T218I, L234I, A265V, R269K, and S283G) in the CTD. Of these six positions, L234I and S283G are highly polymorphic in HIV-nonB. V201I is highly polymorphic in both HIV-1B and non-B (**Figure 1**). Amino acid positions 218 and 283 are missing in structures of IN. Hence, the structural impact of PMs at these positions cannot be deduced unambiguously. V201 is at the interface of inner and outer IN molecules (**Figure 5c**), and it participates in a symmetric interaction. Mutation V201I may increase the buried surface area between two IN molecules by a factor of 265 Å^2^, which empirically can account for ∼15 kCal/mol greater binding energy between the two molecules.

In the structures of IN tetramers bound to DNA, amino acid L234 from the outer IN molecules is adjacent to viral DNA. However, it is not within interacting distance. With mutation L234I, although the sidechain is closer to the DNA, it is still far from any sort of interaction with DNA. In the inner IN molecules, L234 is exposed to the solution and does not participate in either protein–protein interactions or protein–DNA interactions.

Amino acid position 265 resides in the SH3-like fold of CTD. We noticed PM A263V in our sequence analyses. In HIV-1B, A/V265 is part of a hydrophobic cluster constructed by V225, V249, V260, A265, and I267 (**Figure 5e**). A critical amino acid residue with respect to INSTI resistance (R263) is part of the loop connecting V260 and A265 (**Figure 5e**). The sidechain of R263 interacts with the phosphate backbone of viral DNA. Mutation A265V is expected to change the geometry of the hydrophobic cluster, which may affect the interaction of R263 with viral DNA. In fact, our modeled structure of HIV-1C IN shows a slight change in the conformation of R263K, resulting in altered distances of NH1 and NH2 atoms from the phosphate groups of viral DNA.

In the cryoEM structure, K269 is the last residue that could be resolved. Although our sequence analyses show very low R269K PM in HIV-1B, the cryoEM structure contains K269, and it does not interact with the viral DNA. Hence, we modeled R269 in place of K269. The results shown in **Figure 5d** indicate that R269 interacts with the phosphate backbone of the viral DNA molecule (shown as dotted line). While this interaction is not seen with K269, there are two confounding factors associated with this phenomenon. First, since it is the last residue in the solved structure, the conformation of the sidechain cannot be unambiguously deduced. The second factor is that the lysine sidechain is quite flexible, leaving the possibility open that K269 will adopt conformations which can interact with DNA.

**FIGURE 6:**
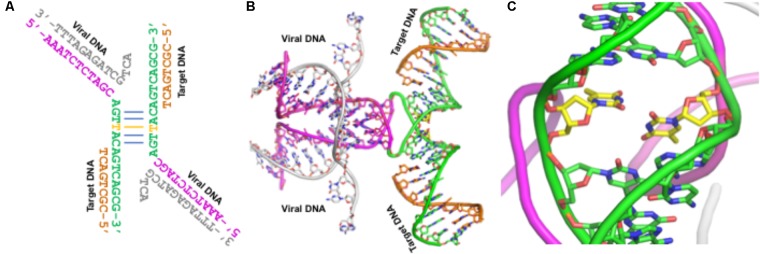
The conformation and sequence of DNA used in the cryoEM structure of IN – Panel **A** shows the viral (gray and magenta) DNA and target (green and orange) DNA. The base-paired region is shown in the middle of this panel. The T–T mismatch in this and in panels **B** and **C** is shown with yellow carbons. The overall DNA conformation is shown in panel **B**. This DNA conformation represents the structure after the strand transfer reaction. Panel **C** shows an enlarged segment from panel **B**.

The cryoEM structure of the HIV-1 STC is a milestone in the structural biology of integrase. By modeling the IN nucleoprotein complexes from different subtypes in combination with sequence analyses, we have elucidated structural aspects and potential functional impacts of IN PMs. However, our analyses and the conclusions drawn from these analyses should be considered with caution, since there are some unusual features associated with the cryoEM structure of the IN intasome. First, the structure was solved as a fusion protein with Sso7. Whether Sso7 fusion has affected any domain rearrangement of IN remains unknown. Second, the IN construct that was used in structure determination contains an active site mutation (E152Q), although it is highly unlikely that this mutation may have affected the overall structure of the IN/DNA complex. The third and most important feature is that the DNA used in this structure determination contains a T–T mismatch in the double-stranded region (**Figure 6**). This mismatch (shown in yellow carbons in **Figure 6**) may have affected DNA bending, which in turn would have affected spatial oligomerization of IN molecules in the intasome.

In summary, we have presented extensive analyses of IN PMs in the structural context. Our analyses suggest that there are several naturally occurring polymorphisms that may affect the structural stabilities of the IN and vDNA binding, and drug binding propensity. Future biochemical and virological experiments will provide deeper insights into the functional impacts of sequence variations among IN genes from different subtypes. Importantly, these studies will also provide guidance for investigating how naturally occurring polymorphisms can affect treatment response in large real-life cohorts.

## Author Contributions

KS and UN conceived and designed the study. UN conducted sequence analyses. LR and KS conducted structural analyses. GJ and AO provided sequence information on TV cohort. KS, and UN wrote the first draft of the manuscript reviewed by GJ, AO, SS, AS, and LR. All the authors approved the final version of the manuscript.

## Conflict of Interest Statement

The authors declare that the research was conducted in the absence of any commercial or financial relationships that could be construed as a potential conflict of interest.

## Footnotes

1 https://www.hiv.lanl.gov/content/sequence/CONSENSUS/consensus.html

2 https://services.mbi.ucla.edu/SAVES/
